# Epigenetic signals associated with delirium replicated across four independent cohorts

**DOI:** 10.1038/s41398-024-02986-w

**Published:** 2024-07-04

**Authors:** Yoshitaka Nishizawa, Kaitlyn C. Thompson, Takehiko Yamanashi, Nadia E. Wahba, Taku Saito, Pedro S. Marra, Takaaki Nagao, Tsuyoshi Nishiguchi, Kazuki Shibata, Kyosuke Yamanishi, Christopher G. Hughes, Pratik Pandharipande, Hyunkeun Cho, Matthew A. Howard, Hiroto Kawasaki, Hiroyuki Toda, Tetsufumi Kanazawa, Masaaki Iwata, Gen Shinozaki

**Affiliations:** 1 Department of Psychiatry and Behavioral Sciences, Stanford University School of Medicine, Palo Alto, CA USA; 2 Department of Psychiatry, Osaka Medical and Pharmaceutical University School of Medicine, Osaka, Japan; 3 University of Nebraska Medical Center, Omaha, NE USA; 4 Department of Psychiatry, University of Iowa Carver College of Medicine, Iowa City, IA USA; 5 Department of Neuropsychiatry, Tottori University Faculty of Medicine, Yonago-shi, Tottori Japan; 6 Department of Psychiatry, Oregon Health and Science University, School of Medicine, Portland, OR USA; 7 Department of Psychiatry, National Defense Medical College School of Medicine, Tokorozawa, Saitama Japan; 8 Department of Neurosurgery, University of Iowa Carver College of Medicine, Iowa City, IA USA; 9 Department of Neurosurgery (Sakura), Toho University School of Medicine Faculty of Medicine, Sakura-shi, Chiba Japan; 10 Sumitomo Pharma Co, Ltd, Osaka, Osaka, Japan; 11 Department of Neuropsychiatry, Hyogo Medical University, College of Medicine, Nishinomiya, Hyogo Japan; 12 Department of Anesthesiology, Vanderbilt University Medical Center, Nashville, TN USA; 13 University of Iowa, College of Public Health, Iowa City, IA USA

**Keywords:** Clinical genetics, Epigenetics in the nervous system

## Abstract

Delirium is risky and indicates poor outcomes for patients. Therefore, it is crucial to create an effective delirium detection method. However, the epigenetic pathophysiology of delirium remains largely unknown. We aimed to discover reliable and replicable epigenetic (DNA methylation: DNAm) markers that are associated with delirium including post-operative delirium (POD) in blood obtained from patients among four independent cohorts. Blood DNA from four independent cohorts (two inpatient cohorts and two surgery cohorts; 16 to 88 patients each) were analyzed using the Illumina EPIC array platform for genome-wide DNAm analysis. We examined DNAm differences in blood between patients with and without delirium including POD. When we compared top CpG sites previously identified from the initial inpatient cohort with three additional cohorts (one inpatient and two surgery cohorts), 11 of the top 13 CpG sites showed statistically significant differences in DNAm values between the delirium group and non-delirium group in the same directions as found in the initial cohort. This study demonstrated the potential value of epigenetic biomarkers as future diagnostic tools. Furthermore, our findings provide additional evidence of the potential role of epigenetics in the pathophysiology of delirium including POD.

## Introduction

Delirium is a significant burden, especially among elderly populations, and it is particularly common after infection or surgery, yet, it remains underdiagnosed and undertreated [1–3]. The consequences of delirium cannot be underestimated including long-term cognitive decline [4] and high mortality [5]. Thus, it is important to develop reliable methods to detect delirium. One approach is to use biomarkers based on molecular signals from patient samples such as blood [6–9]. It has been reported that inflammatory markers tend to be elevated in blood samples obtained from delirium patients [6–9]. Our group previously proposed the epigenetics hypothesis of delirium suggesting that age-associated DNA methylation (DNAm) change in relevant genes can alter the gene expression leading to the pathogenesis of delirium, such as heightened neuroinflammation and decreased neurotrophic processes, and data previously published supported such mechanisms [10–14]. Those data showed the potential usefulness of the molecular signals as a potential biomarker of delirium including POD [11–13].

Our previous report using Gene Ontology (GO) and Kyoto Encyclopedia of Genes and Genomes (KEGG) analysis indicated that similar pathways are enriched both from general inpatient samples and from the neurosurgery samples. Both cohorts showed the same pathways such as immune response and inflammatory pathways, consistent with the potential mechanism of delirium [11, 15]. In addition to those enriched pathways, we have previously identified several genome-wide DNAm differences from the initial inpatient cohort (EOD1 cohort) [11]. However, the data was derived from diverse inpatients with various medical conditions, and the top signals of EOD1 cohort (Table 2) are not readily generalizable to other populations such as patients with POD. Without reliable replication, such signals cannot be utilized as a useful disease biomarker. Thus, to investigate the replicability and reliability of the original top hit DNAm signals in EOD1 cohort, in this report, we analyzed three additional replication cohorts (two from Midwest US, one from Japan), another independent inpatient cohort (EOD2 cohort), one neurosurgery cohort (NSG cohort) we previously analyzed, and one Japanese cohort with various gastrointestinal surgery types (TSG cohort). We specifically hypothesized that genome-wide DNAm signals associated with delirium discovered from the original cohort (EOD1) have similar changes between cases of delirium including POD versus controls.

## Methods

### Study participants

For this study, we included four independent cohorts (Supplementary Table 1). Two cohorts from inpatients, and two cohorts from surgery samples. Three cohorts (two inpatients: Epigenetics of Delirium (EOD1 and EOD2) cohort and one surgery: neurosurgery (NSG) cohort) were recruited at the University of Iowa Hospitals and Clinics (UIHC). Another surgery cohort (Tottori surgery (TSG) cohort) was recruited at the Tottori University Hospital in Japan. Previous reports have described the details of the study participants and the recruitment procedure at the UIHC [11–14, 16, 17]. In short, we enrolled subjects who were either admitted to the hospital between November 2017 and March 2020 (EOD1 and EOD2), or scheduled for brain resection surgery due to their medication-refractory epilepsy at the University of Iowa Hospitals and Clinics between April 2015 and July 2019 (NSG). The recruitment process at the Tottori University was similar, although patients were recruited from gastrointestinal surgery services including gastric, colorectal, and pancreatic surgery between July 2017 and April 2018 (Table 1). We also obtained written informed consent from all participants. All study procedures were conducted with appropriate approval by the Institutional Review Boards from each institution. The authors assert that all procedures contributing to this work comply with the ethical standards of the relevant national and institutional committees on human experimentation and with the Helsinki Declaration of 1975, as revised in 2008. All procedures involving human subjects/patients were approved by Hawk IRB ID #201708758, and the Clinical Research Review Committee of Tottori University Hospital (Reference No. 1704B007).

**Table 1:** Demographic and clinical characteristics of study participants from four cohorts.

Cohort	EOD1			EOD2			NSG			TSG		
Factor	Delirium	Control	*p*-value	Delirium	Control	*p*-value	Delirium	Control	*p*-value	Delirium	Control	*p*-value
*n*	43	44		44	44		10	27		7	9	
Age (years)	70.5	69.9	0.457^a^	78.6	78.6	0.988^a^	41.0	29.8	0.046^a^	81.6	80.1	0.613^a^
SD	10.7	9.8		6.7	7.5		15.6	14.3		6.4	4.2	
Sex (female) %	13	14	0.825^b^	16	17	1.000^b^	5	8	0.444^b^	2	3	1.000^c^
	30	32		36	39		50	30		29	33	
DRS	16.3	6.1	<0.001^a^	12.7	5.4	<0.001^a^	NA	NA	NA	11.4	3.6	<0.001^a^
SD	6.3	3.8		5.8	2.7					3.6	2.2	
DOSS	4.2	0.4	<0.001^a^	5.9	0.3	<0.001^a^	NA	NA	NA	NA	NA	NA
SD	3.3	1.6		2.8	1.1							
Dementia^d^	11	2	0.001^b^	18	3	0.001^b^	0	0	1.000^b^	2	1	0.550^c^
%	26	5		41	7					29	11	

Age and sex were not statistically different between cases with delirium/POD or control without it except among NSG cohort.

^a^Computed using a two-sample *t*-test.

^b^Computed using a chi-square test.

^c^Computed using a Fisher’s exact test.

^d^Dementia among the TSG cohort was defined as MMSE ≤23 before surgery.

### Clinical assessment and data gathering

Medical and surgical history and demographic information were collected from hospital electronical records as well as through the interview of the study participants and their family members when available. In the case of EOD1 and EOD2 cohorts, CAM-ICU [18] positive, Delirium Rating Scale (DRS) [19] >18, or The Delirium Observation Screening scale (DOSS) [20] >3, or a medical record describing delirium, encephalopathy, altered mental status or confusion were categorized as positive case of delirium. In case of NSG cohort, careful chart review of medical records including physical, neurological, and mental status exams as well as nursing reports were conducted to identify delirium cases [21]. If patients were noted to have altered mental status with fluctuations in alertness and orientation, patients were categorized as positive case of POD. For EOD cohorts and NSG cohorts, when it was not clear for the case category, a board-certified consultation-liaison psychiatrist (G.S.) reviewed the case for the final decision on delirium categorization. In the case of the TSG cohort, psychiatrists (T. Y. and T. N.) examined patients, and identified postoperative delirium using the Diagnostic and Statistical Manual of Mental Disorders, Fifth Edition (DSM-5) of the American Psychiatric Association. The Delirium Rating Scale-Revised (DRS-R)-98 was also used to gauge the degree of postoperative delirium [19].

### Blood sample collection and processing

Whole blood samples were collected with EDTA tubes during their hospital stay for EOD cohorts and before and after surgery for NSG and TSG cohorts. For NSG cohorts, blood samples were obtained before the start of surgery as a “pre” sample and at the end of surgery as a “post” sample in the operating room (OR). Immediately after sample collection, blood samples were brought into the laboratory and were stored at −80 °C until downstream DNA extraction and DNAm analysis as a batch. TSG cohort blood samples collected at post-operation day 1 (POD1) were processed for PBMC and stored in the laboratory until later shipped to the University of Iowa and were analyzed for DNAm status.

### Epigenetics analysis for DNA methylation

DNA extraction from whole blood was conducted using the MasterPure DNA extraction kit (MCD85201, Epicenter, Madison, WI, USA) following the manufacturer’s recommended protocol. NanoDrop spectrometry and the Qubit dsDNA Broad Range Assay Kit (Q32850, ThermoFisher Scientific, Waltham, MA, USA) were used to assess the quality of DNA and quantify them. For each sample, 500 ng of DNA was bisulfite-converted with the EZ DNA Methylation Kit (D5002, Zymo Research, Irvine, CA, USA). Genome-wide DNAm status was assessed using the Infinium HumanMethylationEPIC BeadChip Kit (WG‐317‐1002, Illumina, San Diego, CA, USA). The Illumina iScan platform was used to scan the array.

Raw DNAm data was processed using the R packages ChAMP and Minfi. While the four cohorts were filtered separately, the same set of scripts was used for each analysis to ensure quality control procedures were consistent. When raw data was loaded, probes with certain criterion were excluded, such as (i) a detection p-value > 0.01, (ii) <3 beads in at least 5% of samples/probe, (iii) non-CpG, SNP related, or multi-hit probes, (iv) located on chromosome X or Y. Samples normalization was conducted using the beta-mixture quantile dilation, followed by the analysis of differential methylation.

### Statistical analysis

All statistical analysis was managed by R-4.2.1 [22]. The chi-square test or a Fisher’s exact test was used to calculate the categorical data, and a two-sample t-test was used for continuous variables. Estimated cell proportions for CD8 T cells, CD4 T cells, natural killer cells, B cells, monocytes, and granulocytes were calculated by the DNAm Age Calculator available online [23, 24] using the method described in the literature [25]. DNAm differences were calculated with RnBeads based on the limma method [26, 27].

For EOD1 cohort, DNAm levels were compared between delirium cases versus non-delirum controls. Covariates included in the analysis were age, sex, and cell type proportions. For EOD2 cohort, similar comparison was conducted for replication.

For NSG cohort, DNAm levels were compared between POD cases versus non-POD controls in (1) pre-surgery samples and (2) post-surgery samples. For TSG cohort, DNAm levels were compared between POD cases versus non-POD controls in samples obtained from postoperative day 1. Non-adjusted t-test was used when covariate adjustment did not yield statistically significant signals.

### Previous top hit comparison to test replication

Differential methylation analysis of blood samples taken from a previous, independent cohort of hospitalized patients [i.e., epigenetics of delirium (EOD1), n = 87] revealed CpG sites of significant methylation differences between delirium cases and controls [11]. CpG sites were sorted by increasing p-value, with the “top hits” including 13 sites with the most statistically significant differences. These top hit sites of interest were examined among the additional EOD2 cohort, the NSG and TSG cohort comparing delirium versus non-dellirium to investigate potential replication and consistency of those DNAm signals.

## Results

### Participant demographics

#### EOD1

A total of 87 patients who were admitted to UIHC and enrolled in this study with available blood samples for this analysis were identified for age and gender matched set. Among them, 43 patients had delirium, and 44 did not. Average age for delirium group was 70.5 years (SD = 10.7); 13 of 43 (30.2%) were female. Average age for non-delirium group was 69.9 years (SD = 9.8); 14 of 44 (31.8%) were female. There were no significant differences in age between patients with and without delirium as matched pairs (Table 1).

#### EOD2

A total of 88 patients who were admitted to UIHC and enrolled in this study with available blood samples for this analysis were identified for age and gender matched set. Among them, 44 patients had delirium, and 44 did not. Average age for delirium group was 78.6 years (SD = 6.7); 16 of 44 (36.4%) were female. Average age for non-delirium group was 78.6 years (SD = 7.5); 17 of 44 (38.6%) were female. There were no significant differences in age between patients with and without delirium as matched pairs (Table 1).

#### NSG

A total of 37 patients who were scheduled for brain resection neurosurgery and enrolled in this study had available blood samples for this analysis. Their average patient age was 32.8 years (SD = 15.3), 35% were female, and 97.3% were non-Hispanic white per self-report. Among them, 10 patients developed POD (27.0%), and 27 did not (73.0%). Average age for POD group was 41.0 years (SD = 15.6); 5 of 10 (50.0%) were female. Average age for non-POD group was 29.8 years (SD = 14.3); 8 of 27 (29.6%) were female. There were significant differences in age between patients with and without POD (Table 1).

#### TSG

A total of 17 patients who were admitted to Tottori University Hospital and enrolled in this study with available blood samples for this analysis. 1 patient was excluded as an outlier based on the quality control process (Champ QC). Their average patient age was 80.8 years (SD = 5.1), 31% were female, and 100% were Japanese. Among them, 7 patients developed POD (43.8%), and 9 didn’t (56.2%). Their average patient age for POD group was 81.6 years (SD = 6.4), 29% were female. Average age for non-POD group was 80.1 years (SD = 4.2), 33% were female. There were no significant differences in age between patients with and without POD (Table 1). The type of surgery consisted of gastric (n = 5), colorectal (n = 9) and Biliary tract, Pancreas or Duodenum (n = 2).

### Overlap with top hits from previous EWAS of epigenetics of delirium (EOD)

Table 2 shows the top 13 CpG sites with >2% difference in DNAm level identified from our initial EWAS study (EOD1 cohort) comparing delirium inpatients versus those without delirium. We tested these top 13 CpG sites discovered from EOD1 cohort among the additional cohorts including EOD2, NSG and TSG comparing cases versus controls.

**Table 2:**
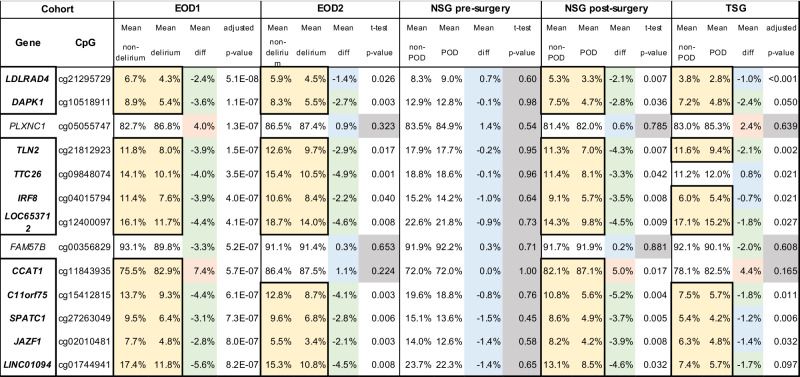
Top hits discovered from the initial EOD1 cohort replicated with additional independent EOD2, NSG and TSG cohorts.

Comparison of DNA methylation level with blood samples between delirium cases versus non-delirium controls among 4 independent cohorts; EOD1 cohort, EOD2 cohort, NSG cohort and TSG cohort. DNA methylation level from blood samples obtained before (Pre-neurosurgery) and after (Post-neurosurgery) surgery among NSG cohorts who did (POD+) and did not (POD−) develop post-operative delirium (POD) are also compared. Adjusted *p*-value: age, gender, and cell type proportions were included as covariates.

Yellow highlights indicate that the DNAm differences are consistent across at least two cohorts.

Green highlights indicate that the DNA methylation level in the case is lower than that in the control (difference is larger than 1.5%).

Red highlights indicate that the DNA methylation levels in the case are higher than those in the control (difference is larger than 1.5%).

Blue highlights indicate CpG sites where the DNAm differences are 1.5 or less.

Gray highlights indicate that a p-value is larger than 0.1 comparing DNA methylation levels between cases and controls.

*diff* difference between case and control about proportion of DNAm, *EOD* epigenetics of delirium, *NSG* neurosurgery, *TSG* Tottori surgery, *POD* post-operative delirium, *LDLRAD4* Low Density Lipoprotein Receptor Class A Domain Containing 4, *DAPK1* Death Associated Protein Kinase 1, *PLXNC1* Plexin C1, *TLN2* Talin 2, *TTC26* The tetratricopeptide repeat domain 26, *IRF8* Interferon Regulatory Factor 8, *LOC653712* Intraflagellar transport 122 homolog (Chlamydomonas) pseudogene, *FAM57B* Family with sequence similarity 57, member B, *CCAT1*: Colon cancer associated transcript 1, *C11orf75* Chromosome 11 Open Reading Frame 75, *SPATC1* Spermatogenesis And Centriole Associated 1, *JAZF1* JAZF zinc finger 1, *LINC01094* Long Intergenic Non-Protein Coding RNA 1094.

**Fig. 1:**
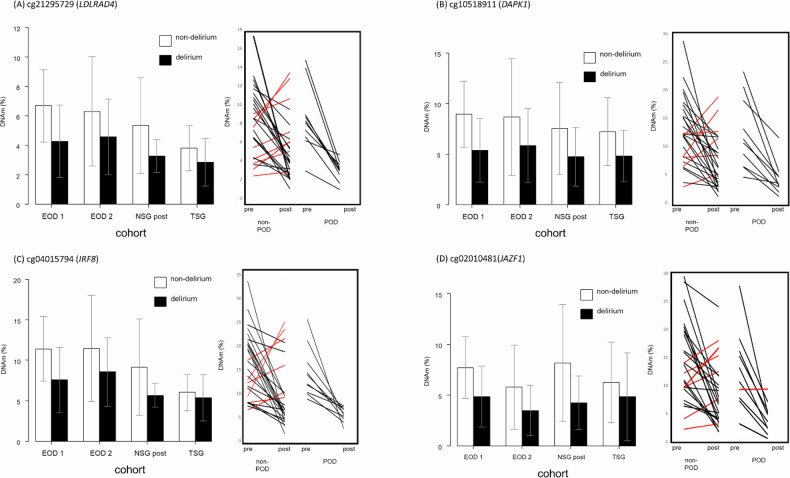
Bar graphs showing DNAm average and linear graphs showing change of DNA methylation levels from pre-surgical to post-surgical blood of each patient. **A** cg21295729 (*LDLRAD4*), (**B**) cg10518911 (*DAPK1*), (**C**) cg04015794 (*IRF8*), and (**D**) cg02010481 (*JAZF1*). (Left) White bars show non-delirium/POD controls and black bars show cases with delirium/POD. Error bars show standard deviation. (Right) Black line: decreased DNAm level from pre-surgery to post-surgery for the NSG cohort. Red line: increased DNAm level from pre-surgery to post-surgery for the NSG cohort. EOD epigenetics of delirium, NSG neurosurgery, TSG Tottori surgery, POD post-operative delirium.

First, EOD2 cohort showed universally similar DNAm level differences between delirium cases versus non-delirium controls. 10 of 13 CpG sites showed difference in DNAm values in the same directions as found in previous EOD1 cohort. Specifically, 10 CpG sites showed lower DNAm levels among delirium cases compared to controls, and levels of difference were nominally significant between the groups as determined by t-tests. Moreover, the degree of DNAm changes were very similar to EOD1 cohort (Table 2, Fig. 1). However, cell count adjustmentmade the result non-significant.

Second, NSG cohort data was tested. When DNAm data from pre-surgery blood samples were compared between POD and non-POD groups, there were no differences across the 13 CpG sites. However, when DNAm data from post-surgery blood samples were compared, similar to EOD2 cohort, 10 of 13 CpG sites showed difference in DNAm values in the same directions as found in previous EOD1 cohort. Specifically, 10 of 11 CpG sites showed lower DNAm levels among delirium or POD cases compared to controls. In addition, one CpG site (cg11843935) showed a higher DNAm level among cases than controls. Again, the levels of difference were nominally significant between the groups as determined by t-tests, and the degree of DNAm changes were very similar to two other cohorts (EOD1 and EOD2) (Table 2, Fig. 1). However, cell count adjustment made the result non-significant.

Of note, when levels of DNAm at these CpG sites were compared before and after surgery, the DNAm levels of patients who developed POD converged to a certain narrow range after surgery, while those from non-POD cases were wide spread (Fig. 1). Moreover, DNAm levels in all of each POD patient pre-surgery was higher than that of post-surgery. although the DNAm level in each non-POD patient pre-surgery wasn’t always higher than that of post-surgery (Fig. 1). This phenomenon was consistently seen across almost all of the CpGs presented here.

Third, TSG cohort data was tested. When DNAm data from post-surgery blood samples were compared, 8 of 13 CpG sites showed difference in DNAm values in the same directions as found in previous three cohorts: EOD1, EOD2, and NSG cohort. Specifically, 8 CpG sites showed lower DNAm levels among delirium or POD cases compared to controls, and levels of difference were nominally significant between the groups as determined by comparison adjusting for age, gender and cell counts. Again, the degree of DNAm changes were very similar between the two cohorts (Table 2, Fig. 1).

## Discussion

We presented data of the epigenetic investigation of delirium using samples obtained from four independent cohorts to test replicability of original top signals identified from inpatient cohort (EOD1). The top CpG sites found from the first cohort was replicated with additional three cohorts, one from similar inpatient cohort (EOD2) and, two from surgery cohorts. Between the surgery cohorts, one consists of neurosurgery patients recruited at the same institution in the Midwestern US (NSG), while the other is from diverse gastrointestinal surgery patients enrolled at Tottori University in Japan (TSG). This is the very first study of its kind to the best of our knowledge reporting replicable epigenetics biomarkers of delirium including POD.

An important finding from this report is strong replication. When we compared top CpG sites from the initial cohort (EOD1) of general inpatients with and without delirium [11], we found significant overlaps in the samples obtained from additional inpatient cohort (EOD2), such as *LDLRAD4, DAPK1, TLN2*, and *IRF8* shown in Table 2. Also, we found the same signals in the neurosurgery patient cohort (NSG). An intriguing fact is that such significant differences were obvious only from post-surgery samples, but not from pre-surgery samples, indicating that those DNAm changes might be unique to delirium status risk, and may not present prior to the development of delirium or POD. Moreover, when we tested post-surgery blood samples obtained from the Japanese surgery cohort (TSG), we identified similar DNAm differences associated with POD. This suggests that the epigenetic signals reported here may be universal and generalizable at least to Caucasian and Asian groups.

Another noteworthy aspect of the present result is that a comparison of pre-surgery and post-surgery in NSG cohorts DNAm level for each patient showed that the DNAm level in each POD patient pre-surgery was higher than that of post-surgery. However, the DNAm level in each non-POD patient pre-surgery wasn’t always higher than that of post-surgery (Fig. 1). In other words, patients with a lower level of DNAm pre-surgery than post-surgery didn’t develop POD for almost all cases.

It is also worth noting that these signals were replicated regardless of the etiology of delirium (such as infection or surgery). Although it is unclear how these genes are involved in the pathogenesis of delirium at this point, these replicated data suggest the reliability of these epigenetic signals as potential biomarkers of delirium from acute illness or surgery. Moreover, our previous analysis of enrichment pathways using data from the same cohorts (EOD1 and NSG cohorts) confirmed the involvement of intriguing and relevant pathways such as immune response, inflammatory response, and cell differentiation from both EOD1 cohort [11] as well as NSG cohort [15].

Several intriguing functions from these top hit genes have been reported in the literature. First, *LDLRAD4* (low-density lipoprotein receptor class A domain containing 4) was one of the top hit genes associated with multiple neurodevelopmental disorders based on the analysis of copy number variations [28]. Second, *DAPK1* (death-associated protein kinase 1) has an important role in regulating apoptosis of macrophages and causing secretion of inflammatory factors [29, 30]. Third, *IRF8* is one of the transcription factors that belongs to interferon regulatory factors. It is also reported that mutation in human *IRF8* has an influence on primarily myeloid cells, and causes immunodeficiency [31]. Lastly, *JAZF1* (juxtaposed with another zinc finger gene 1) reportedly regulates the inflammatory cytokine and inhibits the state of tissue inflammation [32]. Further molecular investigation of these genes and pathways for better understanding of pathophysiological mechanism of delirium would be warranted.

Strengths of this study includes the validation of DNAm signals associated with delirium (1) across four independent cohorts, (2) generalizable findings replicated regardless of etiology of delirium including inpatients as well as post-surgery with diverse surgery types, (3) consistent findings even from across ethnicities and geographic location (Midwestern US and Tottori, Japan).

Also, the timing of blood sample collection for the NSG cohort is noteworthy. The blood was collected at the end of surgery in the OR, before patients were transferred to the recovery suite or hospital floor. Thus, the blood was obtained prior to the emergence of their delirium symptoms. Yet, we were able to see the difference in DNAm signals in their blood. At this point, our current method to measure DNAm takes at least a few days with genome-wide DNAm array approach, and even with alternative approaches such as pyrosequencing, it may not be practical to measure the actual DNAm level soon after sample collection. However, by developing a much faster laboratory technique [33–35], it will become possible to identify those who are at high risk for developing POD and have the ability to intervene before the onset of delirium.

Several limitations in this study should be noted for careful interpretation of the present data. First, although the initial cohort showed genome-wide significant level, the replication cohort did not reach the same genome-wide significance. However, as we did not test other CpG sites across the genome in this specific analysis but rather tested only the 13 top hit CpG sites, genome-wide significance at ~10-8 might be too stringent and cause false negatives. Thus, our findings here can have a certain level of reliability especially given the same directionality and similar level of differences between delirium versus non-delirium groups across four independent cohorts tested. Second, the sample size is small, especially the NSG (n = 37) and TSG (n = 16) cohort, compared to EOD1 (n = 87) and EOD2 (n = 88) cohort. Also for EOD2 and NSG, significant differences were found though t-test, which disappear after adjustment for covariates. Thus, careful interpretation and future replication effort with larger sample size is important. However, even with the smaller sample size, we were able to report similar DNAm signals across these cohorts. Third, in the NSG cohort, younger subjects were included in addition to the older adults. Also, mean age was different between POD and non-POD patients, so we adjusted for age as a covariate to minimize the potential influence of age difference. Fourth, our data does not necessarily indicate the causal relationship between delirium and epigenetics changes. However, as mentioned above, the blood samples of NSG cohort were obtained soon after surgery in the operating room, and it was certainly prior to the onset of POD, suggesting the possibility of such DNAm change playing a role in the pathogenesis of POD. Fifth, our epigenetics data are based on blood samples, and not necessarily reflecting the phenomena in brain. For that effort, we recently reported DNAm status from brain tissues based on our NSG cohort [36]. Such approach would be important to further improve our understanding of epigenetics process in delirium pathophysiology. However, even with these limitations, we observed universally replicable DNAm signals across the four independent cohorts. Sixth, delirium or POD was defined based on clinical assessment both with and without traditional screening tools. Consequently, the definition of delirium cases varies across cohorts, including EOD1/EOD2, NSG, and TSG. However, the definitions of EOD1/EOD2 have been utilized in our previous publications to confirm their validity, as evidenced by substantially higher mortality rates in the delirium group compared to the non-delirium group, aligning well with the well-established fact that patients with delirium have a higher rate of mortality. Additionally, the definition of delirium in TSG adheres to the gold standard set by board-certified psychiatrists. Conversely, the definition of delirium in NSG does not adhere to this gold standard, potentially leading to an increased risk of failing to identify differences in signals between cases and controls. Nevertheless, our data revealed similar signals across all four cohorts, despite the risk of false categorization. Consequently, this conservative assessment is deemed reliable and provides robust signals. Besides, to address the issue of subjectivity in delirium assessment in the current clinical practice as well as in the research methodology, our group has developed a method based on a point-of-care EEG device with a novel algorithm that captures delirium and predicts patient outcomes including mortality, called the bispectral EEG (BSEEG) method [37–40]. If we use such BSEEG tool to assess brain dysfunction in the future studies, we expect that we can discover even stronger signals.

In summary, this is the very first study revealing the epigenetics/DNAm biomarkers associated with delirium (including POD) replicated over four independent cohorts. This data shows the potential usefulness of epigenetics biomarkers as future diagnostic tools, and also points us to additional evidence regarding the potential role of epigenetics in the pathophysiological mechanism of delirium.

### Supplementary information

Supplementary Table 1: Description of each cohort in this study

## Data Availability

The data that support the findings of this study are available from the corresponding author, GS upon reasonable request.
