# Supplementary material for: Epigenetic signals associated with delirium replicated across four independent cohorts
Source: Transl Psychiatry. 2024 Jul 4;14:275. doi: 10.1038/s41398-024-02986-w (PMC11224347; doi:10.1038/s41398-024-02986-w)
Supplement: Supplementary file 1 — Supplementary Table 1: Description of each cohort in this study [file 41398_2024_2986_MOESM1_ESM.docx]

EOD1: A genome-wide DNA methylation (DNAm) case-control association study was conducted. A detailed overview of the recruitment process for study participants has been previously provided (Shinozaki et al., 2018b). In brief, individuals admitted to the University of Iowa Hospitals and Clinics were recruited for this epigenetics study between November 2017 and October 2018. Initially, 92 age- and sex-matched subjects were selected for epigenetics analysis. Among the 92 subjects enrolled, data from 43 delirium cases and 44 non-delirium control subjects were included for analysis after a filtering process.

EOD2：Subjects who were admitted to the University of Iowa Hospitals and Clinics were recruited for this epigenetics study between March 2018 and March 2020. Age and gender matching was performed as in EOD1. After the filtering process, 44 delirium case and 44 non-delirium control subjects were included for the analysis after filtering process.

NSG： The specifics regarding the study participants and the enrollment procedure have been previously outlined (1, 2). In summary, individuals scheduled for brain resection surgery owing to medication-resistant epilepsy at the University of Iowa Hospitals and Clinics between April 2015 and July 2019 were recruited.

TSG：We enrolled subjects who were hospitalized between July 2017 and April 2018. The patients were recruited from gastrointestinal surgery services including gastric, colorectal, and biliopancreatic surgery who provided written informed consent. The inclusion criteria were patients scheduled for surgery for gastric, colorectal, pancreatic, cholangiocarcinoma, and duodenal cancer who were 75 years of age or older at the time of consent. The exclusion criteria were those with schizophrenia, schizoaffective disorder, mood disorders, drug dependence, or alcoholism; and those who, in the opinion of their primary team doctors, were not able to cooperate.
